# Operative treatment of isolated epiphyseal fracture of the distal fibula: 1 case report and literature review

**DOI:** 10.1186/s12891-024-07648-y

**Published:** 2024-07-09

**Authors:** Zhongbo Jiang, Liang Yue, Deheng Wang, Yanchen Liang, Cheng Jing, Yanbo Guo

**Affiliations:** 1 The First Clinical Medical School, Shandong University of Traditional Chinese Medicine, Jinan City, 250355 Shandong China; 2 The Department of Joint Orthopedics, Affiliated Hospital of Shandong University of Traditional Chinese Medicine, 16369 Jingshi Road, Jinan City, 250014 Shandong Province China; 3 The Department of Pediatric Orthopedics, Affiliated Hospital of Shandong University of Traditional Chinese Medicine, 16369 Jingshi Road, Jinan City, 250014 Shandong Province China; 4 The Department of Orthopedics, Affiliated Hospital of Shandong University of Traditional Chinese Medicine, 16369 Jingshi Road, Jinan City, 250014 Shandong Province China

**Keywords:** Isolated epiphyseal fracture of the distal fibula, Syndesmosis, Superior peroneal retinaculum, Ankle fleck sign

## Abstract

Pediatric ankle injuries are common; ankle epiphyseal fractures are also common in children. But isolated distal epiphyseal fibular fractures of the distal fibula are clinically rare. We describe one unusual case of an adolescent with a completely displaced Salter-Harris type II distal fibular epiphyseal fracture. The attempt of closed reduction failed, and the patient required open reduction and internal fixation. The localized periosteum and the superior peroneal retinaculum were avulsed from the distal fibular metaphysis, with the peroneal tendons underneath exposed but no obvious subluxation. To the best of our knowledge, this combination of injuries has not been previously reported.

## Introduction

Epiphyseal injuries are common in children with 12% of fractures of the long bone involving the physis [1], and lower extremity physeal injuries comprised 58.6% of all physeal fractures [2]. Previous studies have reported that physeal fractures make up 18–30% of total fractures [2]. Ankle fractures in children are the third most common fracture involving the growth plate [3]. Ankle fractures in children and adolescents usually are concurrent fractures of the distal tibial and fibular epiphysis. The isolated epiphyseal fracture of the distal fibula is clinically rare in adolescents. The most common fracture type of the distal fibula according to the Salter-Harris (SH) classification are SH type I and II, which are most commonly supination-inversion injuries [4]. The purpose of this article is to highlight a displaced physeal distal fibula fracture that required open reduction due to interposed periosteum and SPR.

## Case report

**Fig. 1:**
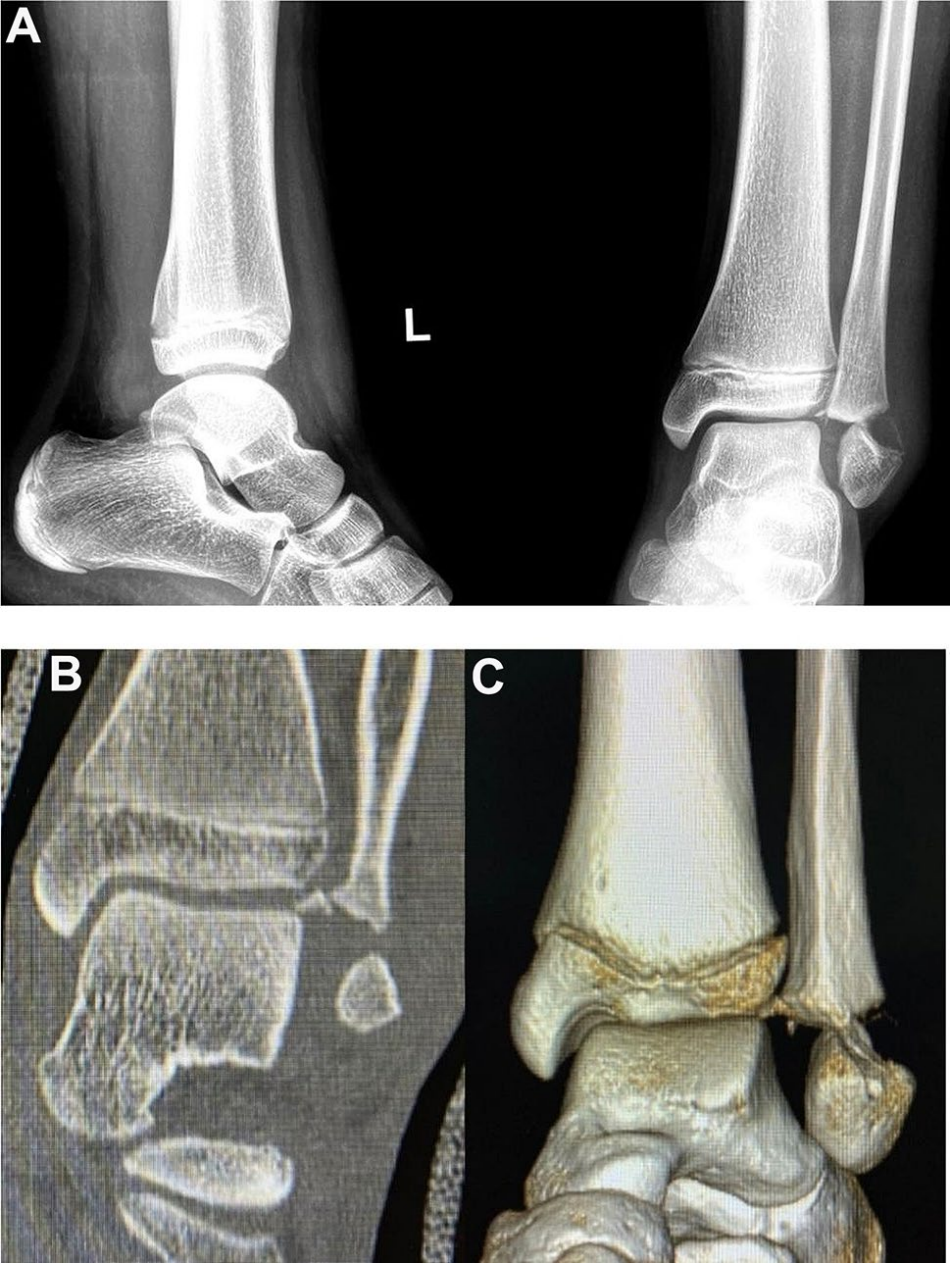
**A**: Standard AP and lateral radiographs taken on presentation to the emergency. **B**-**C**: Typical imaging of coronal CT and 3D images showing the fragment trapped in the syndesmosis

**Fig. 2:**
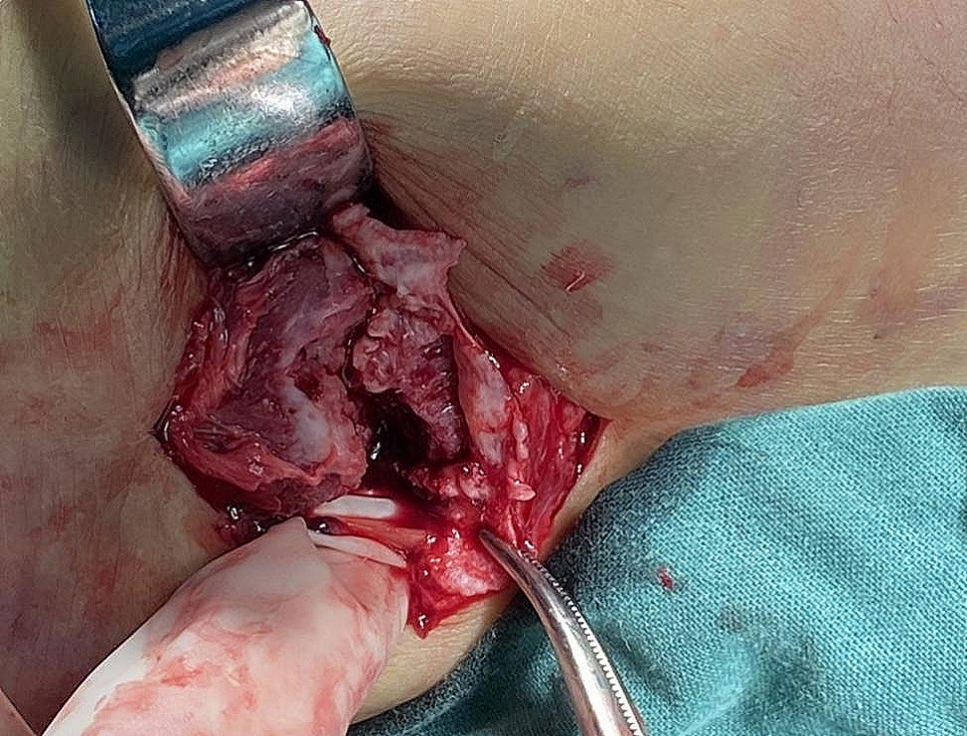
An epiphysiolysis of the distal fibula with a localized periosteum tear and fibular avulsion of the SPR. The peroneal tendons underneath exposed but no obvious subluxation

A 14-year-old male was admitted to the emergency room of our hospital, in May 2021 presenting with left ankle pain after a mechanical fall on the ground. The patient’s personnel and family histories are both unremarkable. Examinations revealed tenderness on the lateral side of the ankle; complete ankle functional impotence and moderately swelling were observed, without any apparent nerve involvement. Standard radiographs revealed a distal epiphyseal fracture of the fibula (Fig. 1). Under epidural anesthesia, the fracture could not be reduced through manipulation. Subsequently, an open reduction and fixation procedure was undertaken using three percutaneous retrograde k-wires. During the operation, the fracture ends were rotationally displaced forward and downward. An epiphysiolysis of the distal fibula was found with a localized periosteum tear and fibular avulsion of the superior peroneal retinaculum (SPR) (Fig. 2). The free fragment trapped in the syndesmosis was removed. The stability of syndesmosis was evaluated by external rotation stress test and hook test, and the torn periosteum and superior peroneal retinaculum were repaired after reduction and fixation.

**Fig. 3:**
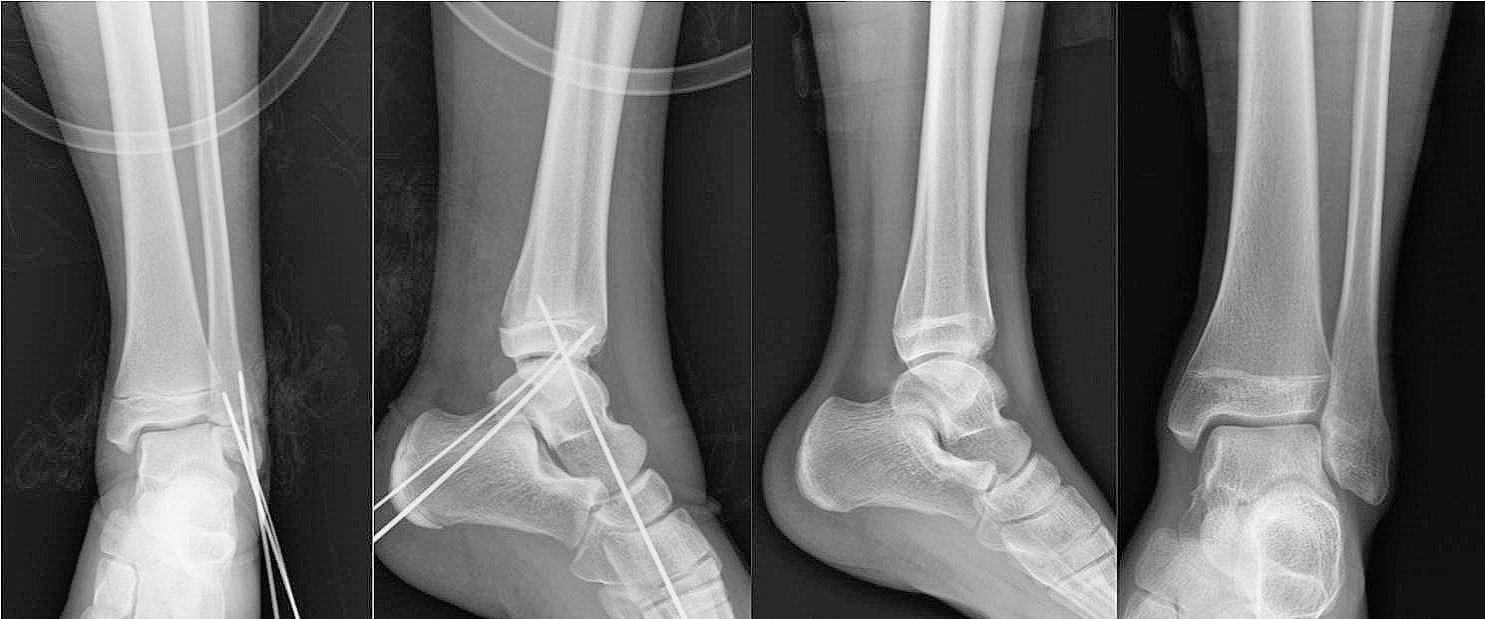
AP and lateral radiographic views of the left ankle during surgery confirming reduction after open reduction and internal fixation and 6 months postoperatively

After surgery, the patient was immobilized with a below-knee cast for 6 weeks. X-rays taken at the end of this period showed healing of the fracture, leading to the removal of the cast and Kirschner pin. The patient successfully returned to his pre-injury level of activity 12 weeks later. At 2 years follow-up, the patient was able to perform full range of motion without any restriction and engage in high-intensity exercises without any pain or discomfort, indicating satisfactory recovery of his ankle function without any complications (Fig. 3).

All procedures undertaken in this current case were conducted in accordance with the ethical standards of the institutional and/or national research committee(s) and followed the Helsinki Declaration (as revised in 2013). Written informed consent was obtained from the patient for publication of this case report and accompanying images. The reporting of this case report conforms to CARE guidelines [5].

## Discussion

Isolated pediatric lateral ankle injuries are common orthopaedic sports-related injuries, mainly including ankle sprain and nondisplaced SH type I distal fibular fracture [6]. Reviewing the literature, there were only five reported cases of isolated epiphyseal fractures of the distal fibula (Table 1). The first case reported as early as 2010 described a SH type IV fibular epiphyseal fracture [7], which was a triplane fracture of the distal fibula. This represents a specific stereotype fracture pattern because of the asymmetric physeal closure and it’s proof that transitional fractures can occur also in locations other than the distal tibia. After this, two cases were reported in 2013: one was a SH type IV fracture with a fragment of the distal fibular metaphysis. The fragment was rotated and incarcerated in the distal tibiofibular joint causing syndesmotic diastasis [8]. The other was a SH type III fracture in which a displaced epiphyseal fragment was wedged in the syndesmosis. The authors [9] postulated that the mechanism of injury was a supination-internal rotation injury, which could be thought of as a reverse Tillaux-type injury. Under the strongly internally rotated anterior-inferior tibiofibular ligament (AITFL), there was causing an avulsion fracture of the distal fibular epiphysis.

**Table 1:** Case reports of isolated epiphyseal fractures of distal fibular

Author (Year)	Case no.	Sex	Age	Side	Trauma	Mechanism	Type	Complications	Management	Follow-up	Outcome
Thomas Ruffing (2010)	1	girl	14	R	falling off a horse	transitional or triplane fracture	SH-IV	none	ORIF with K-wires	5months	Satisfactory
A. Lugeder (2013)	1	boy	9	R	fall while riding scooter and twist	-	SH-IV	fibular avulsion fracture of the AITF ligament	ORIF with K-wires and cerclage fixation	3 years	Satisfactory
N.A. Johnson (2013)	1	girl	12	R	fall awkwardly on a trampoline	an inverted ankle causing a supination-internal rotation injury(a reverse tillaux type injury)	SH-III	syndesmotic diastasis	ORIF with K-wires	3 months	Satisfactory
Jeremy Korsh (2017)	2	boy	14	L	falling to right side while an opposing player stepping on his left foot, causing it to invert forcefully	-	SH-I	avulsion of the AITFL and PITFL with periosteum	ORIF with the plate and screw construct	1 year	Satisfactory
		boy	16	R	sustaining an American football injury	-	SH-I	none	ORIF with threaded cannulated screw	6 years	Satisfactory
Yanbo Guo (presenting case)	1	boy	14	L	fall on the ground after running and jumping	-	SH-I	fibular avulsion of the SPR with periosteum	ORIF with K-wires	2 years	Satisfactory

Displaced epiphyseal fractures of the distal fibula are even less commonly encountered. An article reported two of this type of fracture, both of which were completely displaced SH type I fractures [10]. As the closed distal tibial physis or the child is close to skeletal maturity, the Surgeon chose different fixation strategies with a one-third tubular plate or cannulated screw. In our case, the patient had a completely displaced SH type II distal fibula epiphyseal fracture with a localized periosteal tear and avulsion trapped between the joint space. Fortunately, preoperative imaging showed that the medial clear space was essentially normal. During the operation, the integrity and stability of the syndesmosis were assessed, and the ankle mortise was confirmed to be in good condition. The occurrence of isolated and displaced distal fibular epiphyseal fracture was closely related to the age of the patient, or more accurately, the degree of closure of physis. Transitional fracture occurs during the transition between adolescence and skeletal maturity (11–12). The type of ankle transitional fracture is determined by the ankle injury mechanism and the degree of distal tibial and fibular epiphyseal closure. When the distal tibial physis were nearing complete closure, the energy propagated through the distal fibular physis causing the isolated fibular epiphyseal fracture [10]. What’s more, this type of fracture has a close association with syndesmosis. These factors often influence the choice of surgical repair strategies. Studies have shown that in all cases of isolated distal fibula fractures with widened medial clear space (stressed X-ray) on MR, at least two ligaments-usually the deltoid and syndesmosis groups were partially or completely torn, wherein the AITFL of the syndesmosis suffered complete interruption [13]. The stability of syndesmosis was assessed in almost all the above cases, demonstrating its significant importance. The treatment differs depending on the stability of the syndesmosis. With adequately conducted treatment, all patients had satisfactory outcomes with good function and no complications.

Two patients attempted closed reduction but failed. Patients in all cases were treated with an open reduction and internal fixation finally. Closed reduction was attempted but failed as in previous case reports. Complete epiphyseal displacement, torn periosteum, and trapped fragment may obstruct fracture reduction in this type of fracture. Of note, a localized periosteum tear and fibular avulsion of the SPR were found during the operation. The peroneal tendon (PT) underneath was exposed and compressed by the rotationally displaced epiphyseal fracture ends, but there was no obvious subluxation of the PT. We hypothesize that the torn SPR and compression of the fracture ends to PT are also critical reasons for the failure of closed reduction. Displaced fibular epiphyseal injuries are clinically rare, and to the best of our knowledge, this type of injury complex has not been previously reported. The SPR originates from the posterior portion of the distal fibula at the level of the ankle joint line and attaches to the adjacent periosteum, under which the PT passes through the posterior groove of the lateral malleolus [14]. The SPR is an important stabilizing structure that restricts the peroneal tendon from subluxation [15–17]. The injury of SPR will cause chronic pain and subsequent instability of the ankle. The “Ankle fleck sign”, a linear flake at the lateral cortex of the distal fibula, is a specific signal of rupture of the SPR and PT subluxation. It can be seen on the preoperative X-ray and during the surgery. The close correlation between completely displaced distal fibula epiphyseal fracture and SPR injury needs to be emphasized and further investigated (18–19). Similarly, routine CT examination was recommended to clarify the condition of fracture displacement, evaluate the distal tibiofibular syndesmosis, rule out occult fractures, and assist surgical planning for better treatment and prognosis.

## Conclusion

This article specifically focuses on the surgical management of isolated epiphyseal fractures of the distal fibula, highlighting the unique challenges posed by the interposed periosteum and SPR. A literature review identified five cases of this fracture type and a comparative analysis was conducted on their characteristics, injury mechanisms, and treatment strategies. Prompt and accurate diagnosis and treatment are paramount for effective management of these injuries. The experiences and lessons learned from these cases provide clinicians and researchers with valuable insights and practical guidance.

## Data Availability

All relevant data are within the manuscript and its additional files. No additional data are available.

## Abbreviations

SH: Salter-Harris
AITFL: Anterior-inferior tibiofibular ligament
SPR: Superior peroneal retinaculum
PT: Peroneal tendon
